# Assessment of quality of life in patients with primary hypothyroidism - the main criterion of treatment effectiveness

**DOI:** 10.1192/j.eurpsy.2021.1721

**Published:** 2021-08-13

**Authors:** O. Pityk

**Affiliations:** 1 Psychiatry, Narcology And Medical Psychology, Ivano-Frankivsk National Medical University, Ivano-Frankivsk, Ukraine; 2 Psychiatry, Narcology And Medical Psychology Department, Ivano-Frankivsk National Medical University, Ivano-Frankivsk, Ukraine

**Keywords:** quality of life, hypothyroidism, nonpsychotic mental disorders

## Abstract

**Introduction:**

Сurrent research suggests that the assessment of the quality of life of patients with somatic and mental pathology should be one of the main criteria for assessing the quality of treatmen. Given this view, one of the valid assessment of the performance of integrated mental health and the effectiveness of treatment should be considered as quality of life, defined by WHO. Leading mental health criteria based on such factors as adaptation, socialization and individualization.

**Objectives:**

The aim was to investigate the quality of life of patients with hypothyroidism with non-psychotic mental disorders. We examined 132 patients with hypothyroidism. The age of patients ranged from 25 to 55 years. The main group included 108 patients with non-psychotic mental disorders, which are dominated asthenia (27.78 %), asthenic- depressive (32.41%) and asthenic-anxiety disorders (18.52%). The control group consisted of 24 patients with hypothyroidism without mental disorders.

**Methods:**

Quality of life was assessed using a questionnaire developed by Mezzich, Cohen, Ruiperez, Liu & Yoon (1999), covering the three main components of quality of life: subjective wellbeing/satisfaction, fulfillment of social roles, external living conditions.

**Results:**

Found a significant difference in quality of life in patients with main and control group. The average in the overall perception of life (sense of satisfaction and happiness in general) in the main group was 5.19±1.15, in the control group 7.50±2.25.

**Conclusions:**

The main conclusion is that patients with hypothyroidism really need psychiatric consultation and treatment should include not only endocrinological influence but neuropsychopharmacological and psychological too.

**Disclosure:**

No significant relationships.

